# New York Pathological Society

**Published:** 1860-02

**Authors:** George F. Shrady


### ART. IV.—*New York Pathological Society.*

Regular Meeting, Dec. 28, 1859. Dr. E. R. Peaslee, Chairman *pro tem.*

(Reported by Geo. F. Shrady, M. D., Secretary.)

### BULLET EMBEDDED IN THE OMENTUM.

Dr. Sands presented a portion of the great omentum, the diaphragm, and a section of the walls of the chest, which were removed from a dissecting-room subject, a Chinaman, who committed suicide by hanging. The point of interest connected with the case was the existence of an iron bullet in the omentum, which had evidently been encysted there for a long time and had taken a very round-about course in arriving at the place it was discovered.

The precise situation of this foreign body was about two inches from the left border of the great omentum, and two inches before the greater curvature of the stomach.

A careful examination was made of the integument in the neighborhood, but no corresponding cicatrix was found. On searching a little further, however, a circular spot was discovered on the right side of the thorax, a little posteriorly, and upon removing the skin in this situation and cutting open the chest, a well-marked cicatrix of the pleura was discovered corresponding to the interval between the eighth and ninth ribs; and on looking still further, another cicatrix was found on the pleural surface of the diaphragm, and still another at a corresponding situation on the surface of the diaphragm; all of these being about the same size. This last-mentioned cicatrix was found to be connected by old adhesions with another in the right lobe of the liver, which in its turn was connected with a furrow in the organ about half an inch in extent,—as if the surface had been grazed by the projectile. It was a curious fact, noticed in the examination made, that the convexity of lung-tissue between the point of entrance of the bullet in the chest wall and its passage through the diaphragm was not injured, there being no evidences of cicatricial tissue present.

Dr. Krackowitzer thought that the patient might have taken an expiration at the time of the injury, and thus the lung was lifted out of the way.

In answer to a question from Dr. Peaslee, Dr. Sands stated, that there were several inches of lung-tissue below the cicatrix in the pleura costalis.

Dr. Bibbins asked if the lung extended below the ninth rib behind during inspiration.

Dr. Clark said, that the lower limb of the lung on the right side for auscultation and percussion anteriorly was the lower border of the sixth rib in front, and the ninth intercostal space or perhaps the top of the tenth rib behind.

### ANEURISM OF AORTA AND DISEASE OF HEART.

Dr. Sands next presented two specimens, one of *aneurism* of the *aorta*, another of disease of heart, both removed from the same person. He was requested on the Monday before to make a postmortem examination of a gentleman who died in one of the hotels, where he was employed as night-clerk. This person at the time of his death was thirty-two years of age. His health had been good up to a year and a half before, since which time he suffered very considerably from cough which was paroxysmal in its character, and at times very distressingly severe. He continued, notwithstanding, to attend to his business until one o'clock of Monday morning, when he was seized with a pain in the chest attended with great distress in breathing. After a good deal of difficulty he succeeded in getting to his room, when a physician was summoned. The Dr. found him complaining of pain in the precordial region, and suffering somewhat from dyspnœa; the pulse was feeble and frequent, the skin warm and moist. Morphine was given to relieve the pain, and up to 5 in the morning he had taken a grain of the drug. At that time when the physician saw him he was more comfortable; but at 7½ o'clock he complained of being worse, and died at 9 A. M.

The *postmortem examination* was made on the following day. The parts examined were the thorax and abdomen. In the chest there was discovered to exist an aneurism of the aorta, and some very curious looking deposit in the tissue of the heart itself. The aneurism was small and sacculated. It arose from the right side of the ascending aorta, and was developed mainly in a lateral direction. The aperture leading into the sac was circular, and about the size of a five-cent piece. The tumor itself was hardly larger than an English walnut; its walls were thin, and its cavity partly filled with coagula. At a point anterior and a little to the right, the sac seemed nearly ready to burst. There were two smaller aneurisms situated near the first, which appeared to be simple recesses in the lining membrane of the artery. The lining membrane of the aorta were very red, roughened, and corrugated, and the vessel studded with patches of atheroma. On incising the left ventricle to open the aorta, a small quantity of fluid escaped, which looked like pus; this was found to proceed from a yellowish deposit in the substance of the left ventricle commencing near the situation of the aortic valves, and extending about an inch along the ventricular wall. A portion of the substance was as hard as fibrous tissue; while another was almost fluid in consistency, and had the appearance to the naked eye of pus. Examined under the microscope, however, no pus was found, but it seemed to consist almost entirely of granular matter, being for the most part fatty; besides this there were oil drops present of considerable size, together with a very few fibres. On making a little more careful examination of the cavities of the heart, there was discovered another deposit of a similar nature in the septum of the auricles, thickening it to the extent of about ¾ of an inch, and forming three flattened projections into the cavity of the left auricle. The lining membrane of the heart was absent from these masses, the surface being rough and vascular, as if from superficial ulceration.

Dr. Sands observed, that the specimen was interesting in connection with the suddenness of the man's death. The aneurism was so situated and of a such a size as scarcely to cause death by pressure, and being on the right side it was quite out of the way of the recurrent nerve. In consulting authorities he had found several cases recorded in which sudden death was attributed to the presence in the muscular tissue of the heart of a morbid deposit similar to that seen in the present instance; and he therefore suggested the possibility of such an explanation in reference to the case now under consideration.

Dr. Finnell had met with two specimens of fibroid deposit of the heart, so called, in both instances in old people, and in both the death was sudden. The deposit in each case was in the substance of the left ventricle, and measured three lines in thickness. No other lesion of the organ was discovered.

Dr. Clark remarked: In regard to the cause of death in these cases, there is a fact connected with aneurism that had better be recorded with the case. He had met, he could not say how many, but can now recall three or four instances, where an aneurism has made a certain amount of progress, and the patient has suffered more or less inconvenience from it, and yet it has not appeared to be threatening so far as the symptoms would enable us to judge, when death would occur quite suddenly, and to lead to the apprehension that the aneurism had burst.

On postmortem examination it was found that no such thing had occurred, but that it was just ready to open.

In one instance that he distinctly remembers, it was about to open in the trachea, and there was a little ulceration already begun at that point, but not a drop of blood escaped. His impression is that the action of these ulcers, by which an aneurism opens into the trachea, is twofold,—through the mucous membrane outward toward the aneurism, and from the aneurism inward toward the tube. In another instance the same thing occurred in that case, which was an aneurism of the aorta, the sac was so near being opened that two little drops of blood lay directly under the serous surface covering the pleura. Another case comes to his mind which he saw with Drs. Crane and Wood, where previous to death there was great prostration as if the aneurism had burst; but on postmortem examination there was found to be but a moderate exudation of blood upon the mucous surface of the intestine, entirely unconnected with the aneurism, but not enough to be fatal.

### SENILE GANGRENE.

Dr. Finnell presented a specimen of senile gangrene, taken from a patient of Dr. Thebaud, who removed the limb just below the knee joint. The patient was a gentleman about sixty years of age, and had been afflicted with the difficulty for several months. The anterior and post-tibial arteries were traced down as far as the ankle joint, but no obstruction was found to exist, though the walls of both these vessels were covered with atheromatous deposit. Beyond the ankle joint no dissection was made. The parts involved were the three larger toes with a portion of the internal margin of the foot. The line of demarkation was complete. At the time of the operation, Dr. Finnell searched carefully for the femoral artery of the left side (the left foot being the diseased one), and it could be felt beating very feebly, the slightest pressure would entirely arrest it; yet after the removal of the limb the vessels bled quite freely.

Dr. Peaslee thought, that the disease might have commenced in the capillaries, which probably were in a state of fatty degeneration. In as much as the immediate cause of gangrene was stagnation of the blood, the efficient cause may be found in the heart, arteries, or capillaries of the part.

Dr. Clark supposed that in all cases of dry gangrene the circulation of the part was arrested; while in the humid variety it was necessary that the bloodvessels should be more or less pervious, to supply the parts with the watery element. Hence dry gangrene was more apt to occur at the extremities of the fingers or toes. In one case of the former which came under his observation, and which he sent to the late Dr. Kearney Rogers, the trouble was caused by pressure of an osseous growth from the clavicle upon the subclavian artery. Dr. C. further remarked, that not unfrequently in the progress of this disease you seize upon the precise spot where the artery has undergone a change, the deposit producing a murmur as distinct sometimes as those of the heart.

In relation to Dr. Sand's case of gunshot wound, he stated, that there was a surface-production on each side of the diaphragm, most marked on the under surface. It is not so true, said he, of the pleural surface, where the cicatrix was; still, there is a considerable amount of adventitious matter extending two inches or more below the proper cicatrix, and $\frac{3}{4}$ of an inch above that. That appearance leads him to suggest an explanation of some of the doubts and difficulties that have been in our way in the examination of the case. It is a very well known fact, that adhesions of most serous surfaces gradually break up where there is much motion in the parts covered by the membrane, which occasionally when they break up leave shreds just as are seen in this instance. On seeing these shreds the inquiry occurred to him whether the bullet might not have wounded the pleura pulmonalis, passed through a portion of the lung into the diaphragm, and the resulting cicatrix after the lapse of years been removed, as the circulation in these parts is much more active than in the pleura costalis.

That the under surface of the lung has been adherent, is pretty evident from the tags that can be drawn out.

REGULAR MEETING, Jan. 11, 1859. JOHN C. DALTON, Jr., M.D., President, in the Chair.

### ULCER PERFORATING THE FEMORAL VESSELS.

Dr. T. C. FINNELL presented a specimen of a portion of the femoral artery and vein, removed from a man 24 years of age, who died from syphilitic disease in the St. Vincent's Hospital. The patient was admitted on the 24th of Nov. last, in a very prostrated condition, having suffered from the disease three months. He seemed to be rapidly wasting away; was subject to night sweats, a cough, and was unable to retain anything on his stomach. He complained also of great pain about the right groin, where was situated an ulcer occupying a space four inches by six below Poupart's ligament. There was also an ulcer on one of the ring fingers, the second joint of which was laid bare; and in addition to this, he was suffering from phymosis, a concealed chancre, and an offensive discharge from the orifice of the prepuce. The patient was at once placed upon invigorating diet, and he for a time seemed to improve under the influence, notwithstanding the ulcerated surface extended.

About the 20th of Dec., there was noticed a tendency to hemorrhage from the ulcer on the thigh, but by the prompt application of a compress and bandage, the bleeding was arrested. The next day the hemorrhage from the parts was very free, and the House Surgeon was obliged to make pressure upon the femoral artery, while Dr. Finnell was sent for. On arriving at the Hospital, Dr. F. found a slough directly over the femoral artery, from which situation whenever pressure from above was let up a jet of blood would escape. He placed the ligature on the artery just below Poupart's ligament; but the patient being so much prostrated, and having lost so much blood before, gradually sank, and died on the eighth day after.

After death, a portion of the muscles of the thigh which enveloped the vessels of the part, was removed. It was found that the slough had destroyed the artery and vein to the extent of an inch.

For three days in succession before his death, he suffered from very violent chills, which were more severe in character than the Dr. had ever seen before. There was but one ligature applied to the artery, and that was on the proximal side of the wound; still, there was no hemorrhage from the distal side of the vessel. The death, of course, was due to exhaustion.

Dr. CLARK asked why there was no hemorrhage from the vein, if only one ligature was applied.

Dr. FINNELL could not understand why there should not be hemorrhage both from the vein and the distal side of the artery. The artery an inch above the ligature was filled by a firm coagulum, as was also the case with the other divided extremity.

Dr. Clark asked if the chills were not connected with pyæmia—the pus arising from this diseased vein.

Dr. Finnell presumed such to have been the case, though no post-mortem examination was made with a view to establish that point. He stated that the suppurative discharge was immense.

### EXTENSIVE VALVULAR DISEASE OF HEART.

Dr. Conant next presented a specimen of a heart removed on the Monday previous, from a female patient, 35 years of age. The organ was in a very diseased condition. There was a very extensive deposit in the substance of the valves; contraction of the tricuspid and mitral, and also of the semilunar valves of the aorta, together with attenuation and almost destruction of the semilunar valve of the pulmonary artery.

Eleven years previous to her decease, the patient had suffered a severe attack of rheumatism, since which time she had a good deal of trouble and uncomfortable feeling about her heart, and was perpetually plagued with cold extremities. The symptoms were markedly increased from three years ago, when her house caught fire, and she was burned quite severely about the face and hands, besides suffering considerably from fright. She after a time recovered from the effects of the burn. Her husband stated that he had examined the heart, and the symptom as he described it, was a "blowing sound forwards and backward," without any definite intermission.

The patient some few weeks since was taken with pleuritis, attended with considerable effusion in the cavity of the chest, more especially upon the left side.

On post-mortem examination, we found that the cadaver was plump and well developed. Within the abdominal cavity was found from a pint to a quart of serum. The liver was very much enlarged, and was the seat of cirrhosis, presenting the well-marked hobnailed appearance. The kidneys were also very much enlarged, but they were not removed, and consequently no very careful examination was made of them. The left cavity of the thorax was so filled, that on being opened into, it commenced to overflow; the whole amount was estimated at from four to five quarts. The right cavity contained from a quart to a quart and a half of the same fluid. Covering the pleura pulmonalis and pleura costalis, were some shreds of organized plasma, indicating that inflammation of a pretty severe character had existed. The lungs were more or less collapsed, and in the lower part of the left lobe there was a spot of pneumonitis about the size of a small orange. The rest of the tissue of that lung, as well as the organ of the right side, was healthy throughout. The cavity of the pericardium contained about two ounces of fluid, the heart itself being found to be much enlarged, the cavities of the auricles being considerably distended. Part of the tricuspid valve was found to be adherent, and very much thickened, so that the auriculo-ventricular opening was found to be only five-eighths of an inch in diameter. The mitral valve was similarly affected, there being but a mere slit between the two folds. The semilunar valves of the aorta were also much thickened and adherent, a small triangular communication only being left. The semilunar valves of the pulmonary artery were so thin that on being lifted up looked not unlike the meshes of a spider's web. One or two small openings were also noticed to extend through the folds. The husband stated that the patient had been examined by several physicians, but none could detect the beatings of the pulse at the wrist; but they noticed a distinct pulsation in the jugular vein on both sides of the neck. This throbbing of the veins was more marked when she was excited, and after exertion, at which times the skin would present a livid hue. Both auricles were found to be much enlarged, the walls being very considerably thickened.

Dr. Clark suspected that the pulsation in the vein was owing more to the hypertrophy of the right auricle, than regurgitation by the opening in the valves. That amount of disease, said he, in the different valvular structures is quite extraordinary. The aortic and mitral valves are often diseased together, but the addition of the tricuspid is not usually seen.

### SWALLOWING OF A NICKEL CENT.

Dr. Dalton next presented a penny that was swallowed by a child four years of age, the week before last, on Wednesday morning, about 11½ o'clock. The penny was perfectly bright when first passed, it being a new one. The child received no treatment more than one or two doses of castor-oil, which were administered by the parents, and which operated moderately. After the second day no medicine was given; and on the third day the coin was passed, the little patient suffering from no unpleasant symptoms whatever, before or after. Dr. D. inquired as to the composition of the new cent, and was told that it was nickel and copper, in the proportion of one part of the former to three of the latter.

Dr. Clark knew of two instances in which the new penny had been swallowed. On a Sunday afternoon a Dr., said he, came to me and said that he had given his child one of those new pennies, and that it had been swallowed immediately. He asked me what was to be done. I told him wait, and let us see what nickel will do. I looked into the chemical dictionaries, and consulted authorities, but all that I could find was that the nitrate of nickel was poisonous. As to the copper, that had been swallowed so many times, that I had no apprehension about it. I therefore told him that he had better envelop it in sweet-oil and let it run. He did so, and the next day he came to me and said that the coin had gone through in the course of the night. The very afternoon of that day, Dr. Jas. R. Wood met me and said that a patient of his, a little child, had swallowed one of the new pennies. I acquainted him with the results of my investigations in regard to the nitrate of nickle, related to him the case I had just seen, and told him to do the same as the other Dr. I met Dr. Wood shortly after, and found that the penny had passed the next day. In neither of the instances was the penny much changed in appearance. So I take it, when the foreign body is not retained any unusual time, there is nothing to be feared. In answer to Dr. Dalton, he stated that in both instances the passage was made in less than twenty-four hours.

Dr. Conant referred to a little patient of his, who had swallowed one of the nickel cents, and, during two and a half days that it remained in the intestinal canal, there was a very severe diarrhœa, which, however, ceased soon after the coin was expelled. The metal in that case was found to be slightly discolored.

Dr. Elliott referred to two cases that came under his notice. He was called to the first on a Sunday evening, and, at the urgent solicitation of the friends, gave an emetic, but with no benefit. The body was passed on the Wednesday following, three days after it was swallowed. He then gave such articles of diet as he thought would produce large discharges from the bowels, such as potatoes, mush and milk, &c. In the second case, he ordered no medicine, only such articles of food as would produce large stools, and with a like result as in the former case. He learned that the coin was composed of nickel and copper. In consulting authorities he found that the former metal was used as a tonic, but could not find any notice of its having poisonous properties.

Dr. Sayre in that connection referred to a case he had under charge, of a child who had swallowed a glass marble four days before, and it not yet having made its appearance in the stools, he was a little apprehensive of the result, whether it could pass the ileo-cæcal valve as a flat piece of metal would do.

Dr. Dalton did not see why there should be any difficulty.

Dr. Conant remarked that the marble had the advantage of peristaltic action behind, and had only to push itself past the folds of the valve, which were nothing more than duplications of the mucous membrane. He knew of a patient that swallowed a leaden bullet, that went through in twenty-four hours.

Dr. Clark remarked that physicians had often met with cases of biliary calculi as large as the end of the thumb, and twice the size of an ounce bullet, which passed through the intestine without any difficulty.

### ENLARGED SPLEEN AND LIVER.

Dr. Harris presented the spleen and a section of the liver removed from a patient who died in the Floating Hospital on the 16th of last October, from what was supposed to be pernicious intermittent fever. The patient was taken sick five days before he reached quarantine. He had been for ten days at Savannah discharging and taking in cargo; had always been healthy, with the exception of an attack of severe bilious fever several years before, when engaged in business in the South. On arriving at quarantine, and when admitted, he was in a very prostrate condition, although his intellect was clear, and he was able to answer questions intelligently. He stated that in the course of his sickness he had vomited a great deal, had lost strength very rapidly, and had taken a very considerable amount of medicine. The indication, of course, was the use of quinine, and inasmuch as he still vomited, the Dr. was obliged to resort to injections. The material vomited was of a dark color, granulated, and resembled very much the material of yellow fever. It was soon found that the quinine was not absorbed, and that he still continued cold and prostrated. The vomiting continued every half-hour, and seemed to consist very nearly of pure blood blackened by the acids of the stomach. Some blood was also evacuated by the bowels. This state of things continued until the evening of the 16th, 36 hours after admission, when he died.

The post-mortem examination was held the next day. There was nothing peculiar noticed except the condition of the spleen, which, at the time of the removal, it being much enlarged, was so diffluent that a portion fell over the edge of his hand while he was holding it. The liver presented the ordinary color, and was also somewhat enlarged. The Doctor thought the specimens a little remarkable, inasmuch as the patient had only been sick in all for six days and a half.

In this connection, Dr. H. referred to a second case that was received in the hospital at the same time, who had been in Savannah engaged in the same occupation, and remaining in the place the same length of time as the former, and was, at the time of admission, in the same condition as the other, with the exception, perhaps, that he was more insensible. Unlike the former case, however, the quinine administered was speedily absorbed, and soon produced its legitimate effects. The amount of quinine administered during five days, in this case, was some 450 grains. The first patient had no opportunity to take as much.

### SPECIMEN OF PERICARDITIS.

Dr. Thos. F. Cock presented a heart taken from a patient whom he found in the wards of the New York Hospital at the commencement of his term of service on the 1st of January. He was supposed to be a convalescent from pneumonia which was of three weeks' standing. At the first day of my visit, said he, the patient was simply exhibiting dyspnœa and sickness at the stomach. Dr. Cameron, the House Physician, merely told me that he was convalescent, and I passed by after putting my ear to the back of the chest and detecting a mucous rattle at the root of both lungs. The next day the patient appeared more comfortable. On the third day my attention was called to him, and on looking at him a little more narrowly, I saw that his breathing was more difficult and rapid than it had been before. I was induced to examine the front part of the chest, and there found the following symptoms.

The precordial and right hypochondriac regions were both very much distended; there was also fullness of the left hypochondrium. There was dullness on percussion, traceable from the left side over to the right hypochondrium, and on making the examination with the ear, a distinct single-friction sound, could be heard over the heart. This friction sound was traceable over to the right side, almost to the nipple.

The diagnosis was pericarditis, and I supposed it to be much more recent than the specimen will show to have been the case. I thought, moreover, that there was some enlargement of the liver. I was told by the gentleman who made the autopsy, that the usual incision through the cartilage accidentally extended into the pericardium, and that the heart laid mainly beneath the sternum. The pericardium was found to be very much thickened, and contained about a quart of fluid. The heart and pericardium, as attached now, weigh 60 ounces. The character of the lymph deposited is so uncommon, that I thought it worth while to bring it before the Society as a specimen of pericarditis, ancient, probably, with some recent inflammation, this inflammation being exhibited by lymph which has more tenuity at certain parts. At the lower part of the heart it seems to be gathered down and to hang in pedicles like small polypi. The aortic valves hold water very well, and it is fair to suppose that the mitral valve is diseased at all events. I had much rather see the heart entire than settle the point by spoiling the specimen.

